# Healthcare services access during the COVID-19 pandemic among older people: Paola Bertuccio

**DOI:** 10.1093/eurpub/ckac129.411

**Published:** 2022-10-25

**Authors:** P Bertuccio, GP Vigezzi, C Signorelli, A Zucchi, L Cavalieri d'Oro, D Stuckler, L Iacoviello, S Gallus, A Odone

**Affiliations:** 1 Department of Public Health, University of Pavia, Pavia, Italy; 2 Ca’ della Paglia College, Ghislieri Foundation, Pavia, Italy; 3 School of Medicine, Vita-Salute San Raffaele University, Milan, Italy; 4 Bergamo Health Protection Agency, Bergamo, Italy; 5 Brianza Health Protection Agency, Monza, Italy; 6 Department of Social Sciences and Politics, Bocconi University, Milan, Italy; 7 EPIMED, Insubria University, Varese, Italy; 8 Department of Epidemiology and Prevention, IRCCS Neuromed, Pozzilli, Italy; 9 Department of Environmental Health Sciences, IRCCS Mario Negri Institute for Pharmacologic Research, Milan, Italy

## Abstract

The COVID-19 pandemic strongly impacted older people, not only in terms of clinical outcome but also in care provision. Investigating trends of changes in healthcare services access among older subjects during the pandemic, along with studying potential determinants, is of utmost interest to identify the most at-risk individuals. We used data from LOST in Lombardia, a cross-sectional study conducted on a representative sample of 4,400 older adults (aged 65 or more) in autumn 2020. Data were collected about lifestyles, mental health, and access to healthcare services before and during the pandemic. To investigate potential determinants of changes in healthcare access, we presented prevalence ratios (PRs) estimated through multivariable log-binomial regression models. Twenty-one per cent of the participants increased telephone contacts with general practitioner (GP), 9.6% specialist visits for a fee, while 22.4% decreased GP visits, 7.5% ED access, 6% hospitalisations, 12.3% outpatient visits, 9.1% diagnostic exams. The prevalence of the cancellation or delay of medical appointments by the patient's decision was 23.8%, with higher proportions among men, among individuals aged 75 or over as compared to those aged 65-74, and among individuals with a higher self-reported economic status (p-value<0.05). People with comorbidities more frequently cancelled or postponed visits, reduced ED access or hospitalisations. Moreover, individuals with worsened mental health status showed a higher prevalence to cancel or delay visits and to reduce ED access. The decrease in healthcare provision and consultations could result in mortality and morbidity excess. Our results should inform targeted intervention to bridge the gaps and overcome the health inequalities that the pandemic has deepened. Exploring the underlying reasons and determinants for healthcare avoiding or delaying among the most vulnerable groups is crucial for epidemic preparedness and planning future interventions.

